# *Staphylococcus aureus* Strain-Dependent Biofilm Formation in Bone-Like Environment

**DOI:** 10.3389/fmicb.2021.714994

**Published:** 2021-09-07

**Authors:** Fabien Lamret, Jennifer Varin-Simon, Frédéric Velard, Christine Terryn, Céline Mongaret, Marius Colin, Sophie C. Gangloff, Fany Reffuveille

**Affiliations:** ^1^Université de Reims Champagne-Ardenne, Laboratory BIOS EA 4691, Reims, France; ^2^Plateforme en Imagerie Cellulaire et Tissulaire, Université de Reims Champagne-Ardenne, Reims, France; ^3^Service Pharmacie, Centre Hospitalier Universitaire de Reims, Reims, France; ^4^Université de Reims Champagne-Ardenne, UFR de Pharmacie, Reims, France

**Keywords:** MSSA, MRSA, biofilms, prosthetic joint infection, bone microenvironment

## Abstract

*Staphylococcus aureus* species is an important threat for hospital healthcare because of frequent colonization of indwelling medical devices such as bone and joint prostheses through biofilm formations, leading to therapeutic failure. Furthermore, bacteria within biofilm are less sensitive to the host immune system responses and to potential antibiotic treatments. We suggested that the periprosthetic bone environment is stressful for bacteria, influencing biofilm development. To provide insights into *S. aureus* biofilm properties of three strains [including one methicillin-resistant *S. aureus* (MRSA)] under this specific environment, we assessed several parameters related to bone conditions and expected to affect biofilm characteristics. We reported that the three strains harbored different behaviors in response to the lack of oxygen, casamino acids and glucose starvation, and high concentration of magnesium. Each strain presented different biofilm biomass and live adherent cells proportion, or matrix production and composition. However, the three strains shared common responses in a bone-like environment: a similar production of extracellular DNA and engagement of the SOS response. This study is a step toward a better understanding of periprosthetic joint infections and highlights targets, which could be common among *S. aureus* strains and for future antibiofilm strategies.

## Introduction

Environmental parameters such as nutrient availability, oxygen level, dynamic flow, or static culture represent different stresses for bacteria. One adaptive response is the formation of biofilm with various organizations (Bjarnsholt, 2013; Haney et al., 2018). Into the biofilm state, bacteria adopt a different level of metabolic activity and can be phenotypically different from their planktonic counterpart (O’Toole et al., 2000; Hall-Stoodley et al., 2004). Furthermore, biofilm structure and more precisely the biofilm matrix reduces molecule permeability, and a reduced metabolism allows bacteria to escape the immune system and to be tolerant to antibiotics (Flemming and Wingender, 2010; Stewart, 2015). Moreover, the surface nature has a strong impact on the biofilm structure (Feng et al., 2015; Forson et al., 2020). These bacterial communities can grow on both biotic and abiotic supports, such as medical implants (i.e., catheters, pacemakers, or bone and joint prostheses) (Donlan, 2001; Ahmed et al., 2019). Their implantation leads to a host biological response: chronic inflammation, foreign-body response, and host-protein adsorption like fibronectin cover, which also facilitates biofilm development (Fischer et al., 1996; Gibon et al., 2017a,b; Masters et al., 2019). According to the infection site, the proportion of viable adherent bacteria, dead cells, and matrix within the biofilm could be very variable (Nandakumar et al., 2013; Liu et al., 2020). Focusing on bone context, primary periprosthetic joint infections (PJIs) after surgery represent approximately 2% of total joint arthroplasty (Grammatico-Guillon et al., 2012; Springer et al., 2017; Shoji and Chen, 2020). As a consequence, this problem is a major concern with an increasing need of bone and joint prosthesis due to population aging (Levack et al., 2018; Li et al., 2018; Masters et al., 2019; Shoji and Chen, 2020).

*Staphylococcus aureus*, including methicillin-sensitive (MSSA) and methicillin-resistant (MRSA) strains, is mainly involved in PJIs with biofilm development in the periprosthetic environment (Masters et al., 2019; Shoji and Chen, 2020). *S. aureus* biofilm matrix is mainly composed of polysaccharides, extracellular DNA (eDNA), and proteins (Flemming and Wingender, 2010). Among polysaccharides involved in the biofilm, the polysaccharide intercellular adhesin (PIA), also named poly-ββ(1-6)-N-acetylglucosamine (PNAG), is largely predominant (Karygianni et al., 2020). eDNA originates from bacterial lysis or release by live bacteria (Rice et al., 2007; DeFrancesco et al., 2017). *S. aureus* produces considerable amount of proteins in the biofilm matrix such as enzymes or structural proteins, but it is also able to integrate host plasma proteins such as fibrinogen to its matrix (Flemming and Wingender, 2010; Zapotoczna et al., 2015). All these components stabilize the biofilm structures since enzymes such as dispersin B, DNAse, and protease disrupt biofilm by targeting these components (Sadovskaya et al., 2006; Tetz and Tetz, 2010; Lister and Horswill, 2014; Karygianni et al., 2020). MSSA strains produce PIA-dependent matrix mediated by *ica* operon, whereas MRSA strain biofilms are mainly dependent on surface proteins and eDNA (McCarthy et al., 2015). Those differences underline the fact that antibiofilm strategies cannot be universal but need to be adapted according to the bacterial strain and to the biofilm environment.

Thus, biofilm model choice is of utmost importance and has to be relevant and adapted to studied infection conditions (Bjarnsholt et al., 2013; Coenye et al., 2020). It could be suggested that developing new adapted models for each infection site is at least as important as investigating new antibacterial molecules. Models have been developed to improve the clinical relevance for biofilm studies, but to date, there is no existing *in vitro* bone biofilm model (Muthukrishnan et al., 2019). In order to develop new antimicrobial molecules adapted to control PJIs, study of specific bone environment influence on biofilm initiation is needed. Thus, the identification of bone factors affecting biofilms will allow the creation of an *in vitro* bone-biofilm model, close to clinic infection parameters.

Based on preliminary study, some factors were identified to strongly contribute to MSSA adherence and biofilm maturation: anaerobic growth, absence of nutritional molecules (glucose, amino acids), and high concentration of magnesium (Reffuveille et al., 2018). In this present study, we analyzed three different *S. aureus* strain (including one MRSA) behaviors in response to those identified bone microenvironment factors. We evaluated biofilm biomass, live adherent bacteria, matrix composition, and genes expression. A bone-like environment medium was proposed, and specific parameters were identified like influencing conditions for biofilm formation.

## Materials and Methods

### Bacterial Strains and Culture Media

Three commercially available *S. aureus* strains were used in this study: CIP 53.154, SH1000, and USA300. *S. aureus* CIP 53.154 (sensitivity test organism quality control strain for European Pharmacopeia) equivalent to ATCC9144 or NCTC 6571 was first isolated in Oxford, UK, in 1944 and possess the “Set1 gene cluster” (Heatley, 1944). This strain is methicillin sensitive, whereas two mutations are known in *pbp2* gene (Fuller et al., 2005). SH1000 is another methicillin-sensitive strain and originated from 8325-4 strain with *rsbU* gene repaired (Horsburgh et al., 2002). USA300 strain, meanwhile, emerged first as community-associated MRSA in the United States in the late 1990s and become endemic pathogens worldwide. This strain is known to be implicated in osteomyelitis and was first reported in the United States as a cause of skin and soft issue infection (Diep et al., 2008; Tenover and Goering, 2009).

A minimal medium (MM) [62 mM potassium phosphate buffer, pH 7.0, 7 mM (NH4)_2_SO_4_, 2 mM MgSO_4_, 10 μM FeSO_4_) containing 0.4% (w/v) glucose and 0.1% (w/v) casamino acids] was used in all biofilm models. This medium was modified according to the conditions tested: MgSO_4_ concentration was modified by increasing the amount of added MgSO_4_ (10-folds). Experiments under hypoxic conditions were performed using the GenBag system (Biom rieux, Marcy-l’Étoile, France). For all experiments, the absorbance of overnight cultures was measured at 600 nm to dilute the overnight culture in appropriate and fresh medium to obtain an inoculum with a final absorbance of 0.01, except for real-time quantitative PCR (RT-qPCR); overnight cultures were diluted in the appropriate medium in order to get a final absorbance of 0.1. Media containing bacteria inoculated in microtiter plates were incubated at 37°C for 24 h.

### Light Microscopy

Diluted bacteria are inoculated into a 24-well microtiter plate (Corning, New York, NY, United States) (500 μl per well) and incubated for 24 h. Wells were washed twice with distilled water before imaging. Light microscopy images of the well’s bottom was performed by a Zeiss Axiovert 200 M using 20 × objective.

### Crystal Violet Staining

As previously described, biofilm biomass was evaluated by crystal violet staining (Reffuveille et al., 2014). Briefly, inocula were adjusted to absorbance of 0.01, and 500 μl was distributed in a 48-well plate (Corning, New York, NY, United States). After 24 h of incubation, the planktonic growth was evaluated by measuring the absorbance at 600 nm. The wells were gently washed three times with water, and 500 μl of 0.2% crystal violet was added to each well. Plates were incubated for 20 min in the dark at room temperature. Wells were washed three times with water, and 500 μl of 95% ethanol was added to each well. The absorbance at 595 nm was measured to evaluate the amount of biofilm biomass, which is proportional to the absorbance value. Four biological replicates were conducted, and each included three technical replicates.

### Counting Method

To assess the number of live adherent bacteria, inocula were prepared as previously described with an adjusted absorbance of 0.01, and 500 μl was distributed in 24-well plates with a Thermanox^TM^ coverslip (Thermo Fisher Scientific, New York, NY, United States) placed to stay vertically in the well. After 24 h of incubation, the coverslips were washed with minimal medium and transferred to a 15-ml tube containing 2 ml of minimal medium. Biofilm-embedded bacteria attached to coverslips were then detached by placing the tube in ultrasonic bath (40 kHz) for 5 min. A volume of 100 μl from serial dilutions was plated on nutrient agar plates to determine the quantity of initially attached bacteria. The results of counting method are expressed as the ratio of live adherent bacteria on total bacteria (live adherent and planktonic bacteria). Four biological replicates were conducted, and each included two technical replicates.

### Scanning Electron Microscopy

To prepare the samples, inocula were prepared as previously described with an adjusted OD of 0.01, and 500 μl was distributed in 24-well plates on a Thermanox^TM^ coverslip placed at the bottom of 24-well plates (cell culture treated side up). After 24 h of incubation, the coverslips were washed twice in phosphate-buffered saline (PBS), then fixed in 2.5% (w/v) glutaraldehyde (Sigma-Aldrich, St. Louis, MO, United States) at room temperature for 1 h. After two distilled water rinses, biofilms were dehydrated in graded ethanol solutions (50, 70, 90, and 100% twice) for 10 min. Biofilms were finally desiccated in a drop of hexamethyldisilazane (Sigma-Aldrich, St. Louis, MO, United States). After air drying at room temperature, samples were sputtered with a thin gold–palladium film using a JEOL ion sputter JFC 1100 instrument. Biofilms were observed using a Schottky Field Emission Scanning Electron Microscope (JEOL JSM-7900F). Images were obtained at a primary beam energy of 2 kV (SM-EXG65 electron emitter).

### Confocal Laser Scanning Microscopy

To prepare the samples, inocula were prepared as previously described with an adjusted OD of 0.01, and 500 μl were distributed in 24-well plates on a Thermanox^TM^ coverslip placed at the bottom of 24-well plates (cell culture treated side up). After 24 h of incubation, the coverslips were washed twice in PBS and stained with SYTO^TM^ 9 at 1 μM and (i) propidium iodide (Thermo Fisher Scientific, Waltham, MA, United States) at 20 μM to label live and damaged or “dead” bacteria, (ii) SYPRO^®^ Ruby (Thermo Fisher Scientific, Waltham, MA, United States) (v/v) to label proteins, or (iii) wheat germ agglutinin (WGA) associated with the Alexa Fluor^TM^ 350 conjugate (Thermo Fisher Scientific, Waltham, MA, United States) at 100 μg/ml to label PIA with TOTO^TM^-3 iodide (Thermo Fisher Scientific, Waltham, MA, United States) at 2 μM to label extracellular DNAs. Each label was diluted in 0.9% NaCl. After 30 min of incubation in the dark at room temperature, each coverslip was washed two times with PBS and placed in a 24-well Krystal plate with glass bottom (Dutscher, Porvair, United Kingdom) with the biofilm-side lower before observation with confocal laser scanning microscopy (CLSM) (LSM 710 NLO, Zeiss, Germany). Fluorochromes-labeled matrix compounds were imaged and their volume quantified using IMARIS software (Imaris, RRID:SCR_007370).

### RT-qPCR (RNA Purification and Reverse Transcription)

To prepare the sample, inocula were prepared as previously described with an adjusted OD of 0.1, and 1 ml was distributed in six-well plates (Corning, New York, NY, United States). After 24 h of incubation, the wells were washed twice in PBS to discard planktonic cells, and total RNAs were extracted and cleaned up from *S. aureus* biofilms with MasterPure^TM^ RNA Purification Kit (Lucigen, Middleton, WI, United States) in accordance with the manufacturer’s protocol. Total RNAs were reverse transcribed into complementary DNA (cDNA) using a high-capacity cDNA reverse transcription kit (Lucigen, Middleton, WI, United States) following the manufacturer’s instructions. Transcription products were amplified by RT-qPCR using different primers (Eurogentec, Seraing, Belgium) (Table 1) on a StepOne Plus^TM^ system (Applied Biosystems, Villebon-sur-Yvette, France). The SYBR^TM^ Green Master Mix (Applied Biosystems, Foster City, United States) was used for amplification. After a first denaturation step at 95°C for 10 min, RT-qPCR reactions were performed according to a thermal profile that corresponds to 40 cycles of denaturation at 95°C for 15 s, annealing and extension at 60°C for 1 min. Data collection was performed at the end of each annealing/extension step. The third step that consists in a dissociation process is performed to ensure the specificity of the amplicons by measuring their melting temperature (Tm). Data analysis was performed with the StepOne^TM^ Software v2.3 (Applied Biosystems, Villebon-sur-Yvette, France). Target transcript levels (N-target) were normalized to the housekeeping gene transcript levels, and messenger RNA (mRNA) level with the equation N-target = 2.δCt, where δCt is the Ct value of the target gene after subtraction of Ct for the housekeeping gene (Ayciriex et al., 2017). *gyrB* was used as housekeeping gene for CIP 53.154; *rho* was used for SH1000 and USA300 strains.

**TABLE 1:** Nucleotide sequences of primers used for RT-qPCR and efficiency for each primer couple.

Target gene	Sequences		Efficiency
	
	Forward primer (5′→3′)	Reverse primer (5′→3′)	
*gyrB*	CACGTGAAGGTATGACAGCA	ACAACTTGACGCACTTCAGA	2
*rho*	AACGTGGGGATAAAGTAACTGG	TTCACTTCTTCTGCGTTATGGT	1.9
*icaC*	TCGTATATTTACGTGCCATTATATGTG	AAGCAAGGTGTACCAAAAATGAC	1.9
*recA*	ATAGGTCGCCGAGTTTCAAC	GCGCTACTGTTGTCTTACCA	1.9
*lexA*	TCAATATTTTCTACTGCGGTAATAGG	GAAACGATTCATGTGCCAGTT	2
*sarA*	TTTCTCTTTGTTTTCGCTGATGT	TGTTATCAATGGTCACTTATGCTG	2
*rsh*	CGAAACCTAATAACGTATCAAATGC	TGTATGTAGATCGAAAACCATCACT	2
*cidA*	GATTGTACCGCTAACTTGGGT	GCGTAATTTCGGAAGCAACAT	1.8
*nuc*	TCGAGTTTGACAAAGGCCAA	AAGCAACTTTAGCCAAGCCT	1.9
*fnbpB*	AATTAAATCAGAGCCGCCAGT	AATGGTACCTTCTGCATGACC	2.0
*srrA*	AGCAAGTAATGGCCAAGAGG	TAGTTGCCACCTGGATACCA	2.0

### Statistical Methods

The statistical significance of the results was assessed using the exact non-parametric Wilcoxon–Mann–Whitney test for independent samples (GraphPad Prism, RRID:SCR_002798). Differences were considered significant at *p* < 0.05.

## Results

### Different *S. aureus* Strains Basically Harbor Different Biofilm Formation Patterns

**FIGURE 1:**
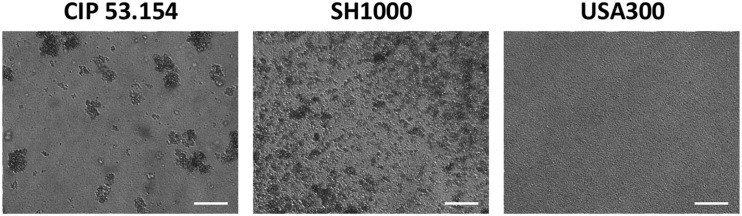
Different biofilm structures according to *S. aureus* strains. Biofilms were grown aerobically for 24 h in minimal medium and imaged by light microscopy. Representative images are shown. The scale bars indicate 40 μm.

In this study, we investigated 24-h-old biofilms to understand initiation of *S. aureus* biofilm formation in early stages of the periprosthetic infection outcome. Three strains of *S. aureus*, naive to bone environment, were selected. Two strains are methicillin-susceptible *S. aureus* (MSSA), namely, CIP 53.154 and SH1000, and the last one is a methicillin-resistant *S. aureus* (MRSA) strain, namely, USA300. Although these strains belong to the same species, their biofilms formed in aerobic condition in minimal medium (MM) presented a different organization, revealed by light microscopy (Figure 1). CIP 53.154 developed three-dimensional aggregates, and only few bacteria were individually adherent between aggregates. The second MSSA strain, namely, SH1000, formed smaller but more numerous aggregates. USA300 displayed a third organization, different from the two previous strains, without any aggregates but a homogeneous colonization of the bottom of the well.

### Impact of Oxygen on Biofilm Biomass and Matrix Formation

**FIGURE 2:**
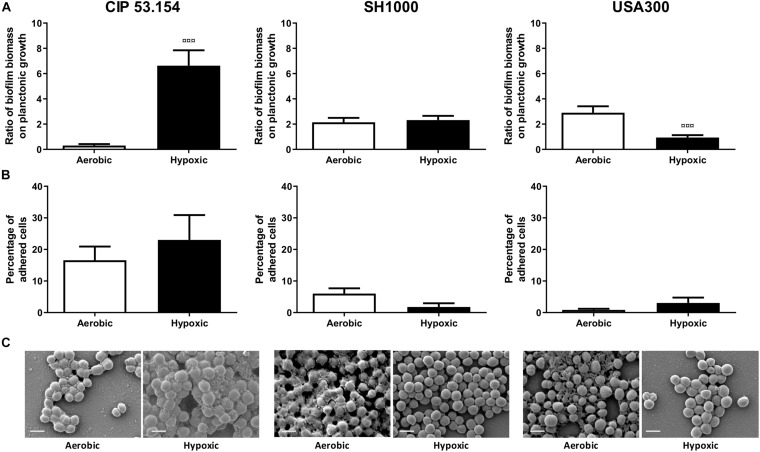
Impact of hypoxia on *S. aureus* biofilm formation. Biofilms were grown in minimal medium with (white histograms) or without (black histograms) oxygen for 24 h before absorbance measurement, live adherent bacteria numeration, and scanning electron microscopy acquisition. **(A)** Results represent the ratio of crystal violet absorbance (biofilm biomass) on planktonic growth absorbance. Experiments were performed at least four independent times with three technical replicates for each. **(B)** Results represent the percentage of live adherent bacteria among all bacteria. Experiments were performed at least four independent times with two technical replicates for each. Error bars represent standard errors for each average value. **(C)** Representative images of scanning electronic microscopy acquisitions at 15,000 × are shown. Statistical analyses were performed using the exact non-parametric Wilcoxon–Mann–Whitney test for independent samples: ¤¤¤Statistically different from aerobic culture condition (*p <* 0.001). The scale bars indicate 1 μm.

The three different strains of *S. aureus* were grown in MM in the presence or absence of oxygen to mimic the hypoxic bone environment (Xu et al., 2016). Both MSSA (CIP 53.154 and SH1000 strains) presented planktonic growth default under hypoxic condition (CIP 53.154, 2.06-fold, *p <* 0.0001; SH1000, 1.57-fold, *p <* 0.0001; Supplementary Material). When cultured with oxygen, CIP 53.154 strain displayed a ratio of biofilm biomass on planktonic growth of 0.3, whereas SH1000 strain presented a ratio of 2.1, and USA300 had the higher ratio of 2.9 (Figure 2A). The three strains differently responded to the lack of oxygen. Indeed, hypoxic condition increased the biofilm proportion formed by CIP 53.154 strain by 21-fold (*p <* 0.001). We did not found statistical difference between the biofilm proportion ratio of aerobic and hypoxic incubation of SH1000 strain (increased by 1.08-fold in hypoxic condition). USA300 strain presented a third behavior: a decrease in biofilm proportion by threefold (*p <* 0.001).

Next, the effect of hypoxic condition on adherent viable *S. aureus* cells was investigated. As seen in Figure 2B, in the presence of oxygen, 16% of viable bacteria were in biofilm state for CIP 53.154 strain, 6% for SH1000 strain, and <1% for USA300 strain. After 24 h of biofilm formation under hypoxic condition, the proportion of adherent viable bacteria was increased by 1.4-fold for CIP 53.154 strain, decreased by 3.4-fold for SH1000 strain, and increased by 3.6-fold for USA300 strain with no statistical differences due to highly dispersed results.

In order to explain results observed with crystal violet coloration and counting method, 24-h-old biofilm samples were observed by scanning electron microscopy (SEM) (Figure 2C). The three strains of this study produced few matrix structures under aerobic culture, sticking bacteria to each other. We observed a different matrix aspect, such as slime or granular structures. Oxygen-depleted culture led to a loss of matrix production for SH1000 strain and MRSA strain, whereas CIP 53.154 strain produced more matrix, which reinforced adherence between bacteria.

**FIGURE 3:**
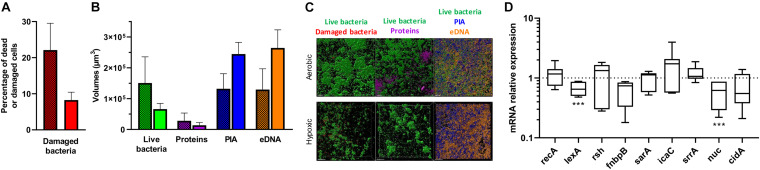
Effect of hypoxia on matrix formation and gene regulation of *S. aureus* CIP 53.154. Biofilms were grown in minimal medium with or without oxygen for 24 h before staining and confocal laser scanning microscopy acquisitions or RT-qPCR analysis. **(A)** Results represent the percentage of dead or damaged bacteria (stained by PI) among live bacteria (stained by SYTO^TM^ 9) acquired by confocal microscopy. Grid histograms represent biofilm growth with oxygen, whereas non-grid histogram represents growth without oxygen. **(B)** Results represent the volume measurement of SYTO^TM^ 9 (green histograms, live bacteria), SYPRO^®^ Ruby (magenta, protein), WGA (blue, PIA), and TOTO^TM^-3 (orange, extracellular DNA) acquired by confocal microscopy. Grid histograms represent biofilm growth with oxygen, whereas non-grid histogram represents growth without oxygen. **(C)** Representative images of confocal microscopy acquisitions are shown. The scale bars indicate 20 μm. Experiments were performed two independent times with three representative acquisitions for each coverslip. **(D)** Results represented relative mRNA expressions of bacteria within biofilms grown hypoxically vs. those grown aerobically. Experiments were performed eight independent times with two technical replicates for each. Results are presented as box and whiskers: whiskers represent minimum and maximum, the bottom and top of the box are the 15th and 85th percentiles, and the black band inside the box stands for the median. Statistical analyses were performed using the exact non-parametric Wilcoxon–Mann–Whitney test for independent samples: ^∗∗∗^Statistically different from aerobic culture condition (*p <* 0.001).

We next focused our attention on CIP 53.154 strain owing to the large increase in biofilm proportion due to hypoxic condition. When cultured with minimal medium and oxygen, 22% of bacteria included in the biofilm were dead or damaged (Figure 3A). Oxygen deprivation led to a decrease in dead and damaged bacteria proportion, which reached 8%. Live bacteria volume reached 1.5 × 10^5^ μm^3^ in aerobic culture and was decreased to 0.6 × 10^4^ μm^3^ for culture without oxygen, without any statistical significance (Figure 3B). Protein-labeled volume represented the lowest matrix component stained (2.8 × 10^4^ μm^3^), and the oxygen deprivation induced a twofold decrease. PIA and eDNA volumes were instead increased by oxygen depletion by 1.8-fold (of 1.3 × 10^5^ μm^3^) and twofold (of 1.3 × 10^5^ μm^3^), respectively (Figure 3C).

To better understand these results, gene regulation was monitored by RT-qPCR analysis. Stress-responses-related genes (*recA*, *lexA*, and *rsh*), adhesion-related gene (*fnbpB*), and biofilm formation and matrix production-related genes (*sarA*, *icaC*, *srrA*, *nuc*, and *cidA*) were thus studied (Rice et al., 2007; Buck et al., 2010; Archer et al., 2011; Geiger et al., 2014; Josse et al., 2017; Mashruwala et al., 2017; Podlesek and Žgur Bertok, 2020). When CIP 53.154 strain was cultured without oxygen, *lexA* and *nuc* were statistically downregulated to 0.65- and 0.62-fold (*p <* 0.001), respectively (Figure 3D). Without statistical difference, *rsh* and *icaC* were upregulated by 1.33- and 1.73-fold, *fnbpB* and *cidA* were downregulated to 0.74- and 0.55-fold. *recA*, *sarA*, and *srrA* displayed no changes in their expression.

### Impact of Bone Condition on Biofilm Biomass, Matrix Formation, and Gene Expression

**FIGURE 4:**
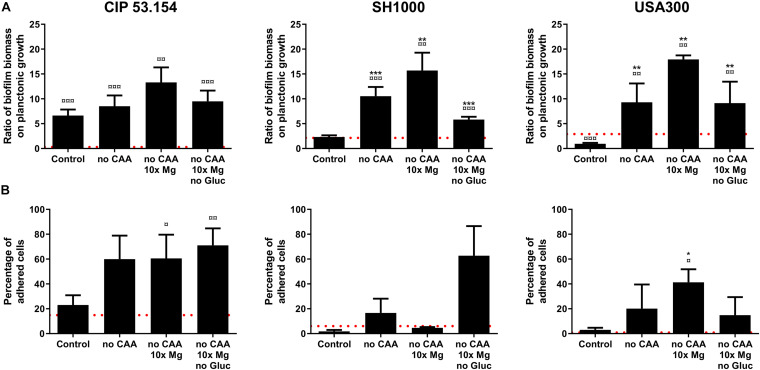
Impact of starvation, magnesium excess, and hypoxia on *S. aureus* biofilm formation. Biofilms were grown in minimal medium (control) or modified minimal medium (no CAA, without casamino acids; 10 × Mg, 10 × minimal medium concentration; no Gluc, without glucose) without oxygen for 24 h before absorbance measurement and live adherent bacteria numeration. **(A)** Results represent the ratio of crystal violet absorbance (biofilm biomass) on planktonic growth absorbance. Experiments were performed at least four independent times with three technical replicates for each. **(B)** Results represent the percentage of live adherent bacteria among all bacteria. Experiments were performed at least four independent times with two technical replicates for each. Error bars represent standard errors for each average value. Red grid line represents results of biofilms grown with oxygen in minimal medium. Statistical analyses were performed using the exact non-parametric Wilcoxon–Mann–Whitney test for independent samples: ¤,¤¤,¤¤¤Statistically different from aerobic control (*p <* 0.05; *p <* 0.01; *p <* 0.001); *, **, ***Statistically different from hypoxic control (*p <* 0.05; *p <* 0.01; *p <* 0.001).

In a second approach, based on a previous study, we modified MM to mimic bone environment parameters (Reffuveille et al., 2018). For this, amino acids and glucose were removed during preparation to mimic starvation, whereas magnesium was added in excess (10-fold initial concentration) to approach the parameters of the bone context. In order to investigate common staphylococcal responses, we selected conditions for which biofilm proportion was similar for all studied strains. In this way, three combinations demonstrated an impact on biofilms: the paucity of casamino acids alone (no CAA) or with magnesium excess (no CAA, 10 × Mg) and a third condition bringing together the lack of amino acids and glucose, with magnesium excess (no CAA, 10 × Mg, no Gluc), which better represented a bone-like environment (BLE). As shown in Figure 4A, casamino acids removal led to an increase in CIP 53.154 strain biofilm proportion by 1.2-fold (*p <* 0.01), SH1000 strain by 4.5-fold (*p <* 0.001), and USA300 strain by 9.9-fold (*p <* 0.01). In addition to the lack of amino acids, high concentration of magnesium reinforced the biofilm proportion, giving the highest biofilm proportion observed for each strain: CIP 53.154, SH1000, and USA300 biofilm proportion were enhanced by 2-fold (*p <* 0.01), 6.7-fold (*p <* 0.01), and 19.1-fold (*p <* 0.01), respectively. The last condition, bringing together the lack of amino acids, glucose, and 10 times the magnesium concentration, increased biofilm proportion of the three strains but in a smaller proportion than the previous condition: CIP 53.154 by 1.4-fold (*p <* 0.001),S H1000 by 2.5-fold (*p <* 0.001), and USA300 by 9.7-fold (*p <* 0.01).

All strains showed an increase in live adherent cell percentages, grown in the three tested conditions (Figure 4B). CIP 53.154 strain initially displayed 23% of bacteria in biofilm state in hypoxic control medium, which increased to 71% in BLE condition. The other MSSA, SH1000 strain, increased its biofilm-embedded bacteria percentage from 1.7 to 62% in BLE condition, whereas MRSA USA300 strain initially displayed 3% of cells under adherent state with control, which increased to 14% in BLE condition.

**FIGURE 5:**
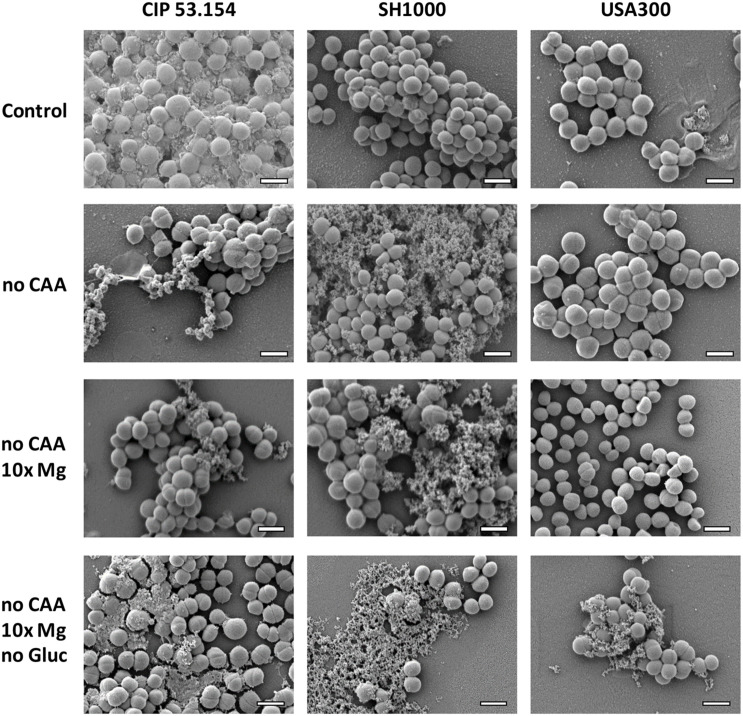
Impact of starvation, magnesium excess, and hypoxia on *S. aureus* biofilm matrix. Biofilms were grown in minimal medium (control) or modified minimal medium (no CAA, without casamino acids; 10 × Mg, 10 × minimal medium concentration; no Gluc, without glucose) without oxygen for 24 h before imaging. Representative images of scanning electronic microscopy acquisitions at 15,000 × are shown. The scale bar indicates 1 μm.

As seen before, when biofilms were grown in MM, CIP 53.154 strain produced an important matrix under oxygen deprivation. SH1000 and USA300 did not seem to produce matrix within the same condition. Casamino acids depletion (no CAA) led to an important decrease in matrix production for CIP 53.154, whereas SH1000 strain produced more matrix, which formed aggregates (Figure 5). MRSA strain USA300 still did not produce any detectable matrix. If magnesium was added in excess (no CAA, 10 × Mg), the aspect and amount of matrix were similar than in the previous condition (no CAA). The last condition bringing together the lack of both amino acids and glucose and magnesium excess (no CAA, 10 × Mg, no Gluc) led to biofilm matrix production for the three strains in variable amount but with a common granular aspect.

**FIGURE 6:**
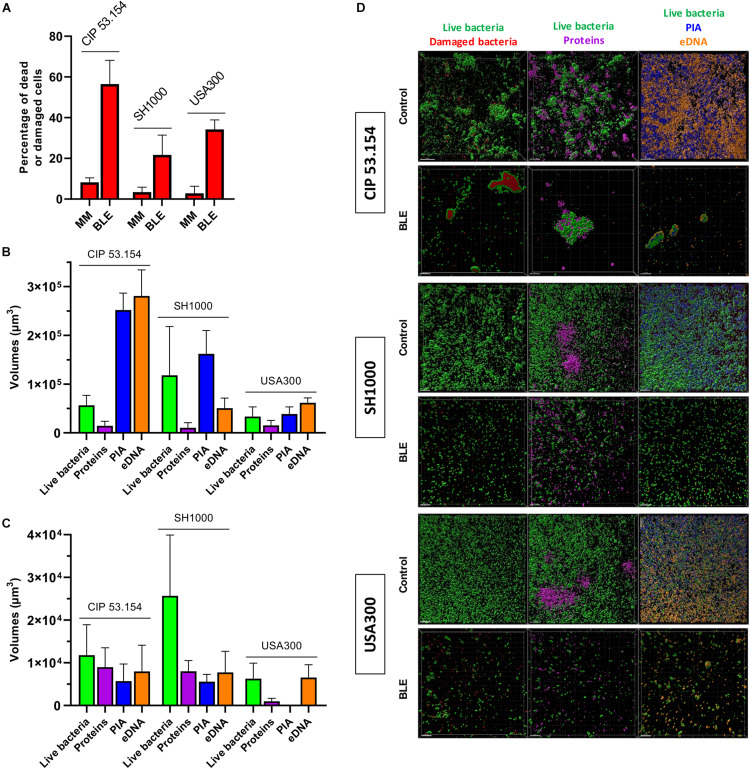
Impact of starvation, magnesium excess, and hypoxia on *S. aureus* biofilm matrix components. Biofilms were grown in minimal medium (MM) or modified minimal medium (BLE: no CAA, without casamino acids; 10 × Mg, 10 × minimal medium concentration; no Gluc, without glucose) without oxygen for 24 h before confocal scanning electron microscopy imaging. **(A)** Results represent the percentage of dead or damaged bacteria (stained by PI) among live bacteria (stained by SYTO^TM^ 9). **(B)** Results represent the volume measurement of SYTO^TM^ 9 (green histograms; live bacteria), SYPRO^®^ Ruby (magenta: protein), WGA (blue: PIA) and TOTO^TM^-3 (orange: extracellular DNA) of bacteria grown in MM without oxygen. **(C)** Results represent the volume measurement as previously described in BLE without oxygen. **(D)** Representative images are shown. Experiments were performed two independent times with three representative acquisitions for each coverslip. Experiments were performed two independent times with three representative acquisitions for each coverslip. Error bars represent standard errors for each average value. Scale bar = 20 μm.

Grown in MM, all strains presented few dead or damaged cells within their biofilms: 8, 3, and 3% for CIP 53.154, SH1000, and USA300 strains, whereas the bone-like environment induced an increase in dead or damaged cells, which reached 56, 21, and 34%, respectively (Figure 6A). As previously mentioned in Figure 3B, the biofilm formed by CIP 53.154 strain in MM and hypoxic culture was mainly composed of eDNA and PIA. SH1000 strain biofilm incubated in MM in hypoxic condition was mainly composed of PIA as volumes of WGA reached 1.6 × 10^5^ μm^3^ (Figure 6B). The second main component was eDNA, which reached 0.5 × 10^5^ μm^3^. We observed opposite results with USA300 strain, as the matrix of this bacterial strain was mainly composed of eDNA (6.1 × 10^4^ μm^3^) and less PIA (3.8 × 10^4^ μm^3^). Incubation with BLE led to the reduction in almost every studied component of each strain. CIP 53.154 strain dramatically modified the composition of its matrix under BLE condition with a slight increase in matrix proteins and an important decrease in PIA (44-fold) and eDNA (35-fold) and proportionally to live bacteria (Figure 6C). Raw volumes of stained matrix components are equal for MSSA strains despite a higher amount of SYTO^TM^ 9 stained volumes for SH1000, whereas USA300 present the same amount of eDNA, but an important decrease in protein amount and almost no PIA was detected (Figure 6D).

**FIGURE 7:**
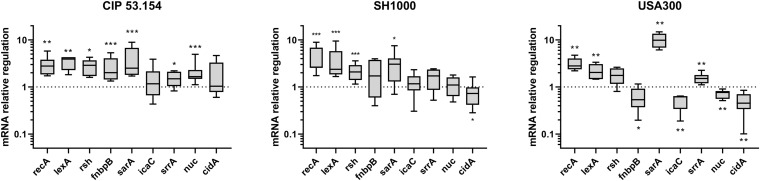
Impact of starvation, magnesium excess, and hypoxia on *S. aureus* gene regulation. Biofilms were grown in minimal medium (MM) or in modified minimal medium (BLE: no CAA, without casamino acids; 10 × Mg, 10 × minimal medium concentration; no Gluc, without glucose) without oxygen for 24 h before RT-qPCR analysis. Results represent the ratio of relative mRNA expressions of bacteria within biofilms grown in BLE vs. those grown in minimal medium. Experiments were performed eight independent times with two technical replicates for each. Results are presented as box and whiskers: whiskers represent minimum and maximum, the bottom and top of the box are the 15th and 85th percentiles, and the black band inside the box stands for the median. Statistical analyses were performed using the exact non-parametric Wilcoxon–Mann–Whitney test for independent samples: *, **, ***Statistically different from minimal medium without oxygen (*p <* 0.05; *p <* 0.01; *p <* 0.001).

Next, we compared gene expressions of bacteria within biofilms grown in BLE vs. MM, both in the absence of oxygen. Four genes were upregulated among CIP 53.154, SH1000, and USA300 strains: *recA* (1.33-fold, *p <* 0.01; 2.64-fold, *p <* 0.001; 2.80-fold, *p <* 0.01), *lexA* (3.92-fold, *p <* 0.01; 2.37-fold, *p <* 0.001; 2.03-fold, *p <* 0.01), *rsh* (2.88-fold, *p <* 0.05; 2.08-fold, *p <* 0.001; 1.30-fold), and *sarA* (2.51-fold, *p <* 0.001; 3.05-fold, *p <* 0.05, 9.87-fold, *p <* 0.01) (Figure 7). Focusing on *fnbpB* relative expression, the three strains behaved differently when cultured in BLE: an increase of 2.01-fold (*p <* 0.001) for CIP 53.154 strain, spread results for SH1000, and a decrease to 0.53-fold (*p <* 0.05) for USA300. Regarding the production of polysaccharides involved in matrix composition through *icaC* and *srrA*, exclusively USA300 strain had a downregulation of *icaC* expression (to 0.62-fold, *p <* 0.01), and *srrA* was upregulated for CIP 53.154 strain (1.49-fold, *p <* 0.05) and USA300 strain (1.51 fold, *p<*0.01) only. Finally, concerning genes involved in eDNA regulation, *nuc* was enhanced by 1.6-fold (*p <* 0.001) for CIP 53.154 strain, remained constant for SH1000, and downregulated to 0.76-fold (*p<*0.01) for USA300 strain; *cidA*, on the other hand, was downregulated for both SH1000 and USA300 strains to 0.72-fold (*p <* 0.05) and 0.45-fold (*p <* 0.01), respectively.

## Discussion

Despite many preventive actions, the number of prosthetic infections cannot decrease without a better knowledge on bacterial biofilm development in bone context. Biofilm structure and composition are dependent on many environmental parameters. Biofilm kinetic and biomass can also vary according to *S. aureus* clonal lineage (Tasse et al., 2018). Methicillin susceptibility is also a key factor, as MSSA and MRSA expressed different levels of exopolysaccharides, protein, and eDNA (McCarthy et al., 2015). Thus, even before being influenced by environmental stress, different strains of the same species form different biofilm structures, probably by responding in a different way to stimuli.

Our data confirmed that different *S. aureus* strains formed various biofilm structures in classic *in vitro* model. Interestingly, even both MSSA exhibited two different biofilm patterns. CIP 53.154 developed a low amount of biofilm but important aggregates, whereas SH1000, a strong biofilm former, conceived microcolonies with an additive adherent monolayer. MRSA strain showed a third response: biofilm grown in monolayer without any cluster.

While *S. aureus* strains are natural biofilm formers, the host microenvironment strongly modulates their biofilm production and, therefore, influences infection establishment with impact on metabolic changes and antimicrobial tolerance of bacteria (Crabbé et al., 2019). Indeed, bone environment factors influence a MSSA biofilm formation (Reffuveille et al., 2018). In the bone microenvironment, the influence of severe oxygen limitation on bacteria was evidenced in *in vivo* models by the enhanced expression of genes involved in fermentation such as *plfB* and *aldA*, supported by high concentration of lactate and presence of ethanol (Xu et al., 2016). In our hands, hypoxic conditions in MM led to a slight decrease in adherent bacteria proportion and a loss in matrix production for SH1000. USA300 also lost its ability to produce biofilm matrix, and the adherent bacteria proportion was slightly increased. However, biofilm biomass proportion formed by SH1000 and USA300 strains was not increased under hypoxic stress contrary to results reported in another study (Mashruwala et al., 2017). We suggested that the way to generate hypoxia, the growth media used and the culture duration could explain such differences, highlighting the importance of biofilm growth model. Only CIP 53.154 strain seemed to significantly produce more biofilm under lack of oxygen, with high production of matrix, mainly composed of PIA and eDNA. PIA is known to be produced by *S. aureus* under oxygen deprivation (Cramton et al., 2001). The eDNA release could be caused by cell wall embrittlement leading to cell lysis (Mashruwala et al., 2017). Interestingly, the CIP 53.154 proportion of dead bacteria included within the biofilm decreased in MM without oxygen. We speculated that 24-h biofilms were in maturation process and are currently under a reorganization phase: lysed cells were evacuated, and eDNA was used for matrix construction, whereas biofilm-embedded bacteria increased their survival rate. The production of accumulated matrix polysaccharides for this strain needs to be investigated.

Focusing on CIP 53.154 biofilm matrix under hypoxic stress, bacterial response was investigated though RT-qPCR study. Due to bacterial phenotype heterogeneity within biofilms and owing to the fact that only a global response of both active and dormant bacteria within biofilm is evaluated, all significant changes were considered. Two genes were significantly downregulated under hypoxic stress compared to aerobic condition: *lexA* and *nuc.* The SOS response (inducer encoded by *recA* and repressor encoded by *lexA*) is triggered by various endogenous factors including starvation through the activation of the stringent response (*rsh* gene) (Podlesek and Žgur Bertok, 2020). SOS response is involved in biofilm formation and FnbpB production, which is responsible for fibronectin attachment (Podlesek and Žgur Bertok, 2020). Under hypoxic condition, a slight increase in *rsh* was noticed. Surprisingly, no *recA* or *fnbpB* induction was observed. On the contrary, we noticed a non-significant decrease in *fnbpB*. We deduced that hypoxic stress induced a global stress response for the establishment of the biofilm in this particular condition. This could explain the non-significant increase in *icaC* expression. This gene codes for PIA, which allow bacteria to adhere and build biofilm matrix (O’Neill et al., 2007; Archer et al., 2011; Nourbakhsh and Namvar, 2016). The *nuc* downregulation could be expected in the early stage of biofilm construction. Indeed, *nuc* is involved in eDNA cleavage and consequently in the inhibition of biofilm initiation (Mann et al., 2009; Berends et al., 2010; Kiedrowski et al., 2011). Its marked dysregulation in the absence of oxygen showed the importance of the biofilm activation under this stress. *cidA*, a gene involved in cell lysis leading to DNA release to build biofilm matrix, was also downregulated (Rice et al., 2007). We speculated that the high eDNA concentration in CIP 53.154 matrix biofilm after 24 h led to a negative feedback control on *cidA* expression or *lgr* genes, marking the end of initiation and structuration of biofilm (Archer et al., 2011). The reinforcement of biofilm in this hypoxic environment underlined the hypothesis that biofilm is a survival strategy.

In conclusion, hypoxic environment differently affected various *S. aureus* strains. While its importance is obvious, this one factor alone could not explain the ability of *S. aureus* biofilm establishment in bone context. These differential results underline the complexity of biofilm and the impossibility to draw universal conclusion on the influence of one environmental factor on biofilm formation even among the same species. In this study, CIP 53.154 strain demonstrated altered quantity of biofilm matrix in response to hypoxia by greatly increasing polysaccharides and eDNA concentration. This modification is probably an accentuated stress response, managed by global systems, due to lack of oxygen.

To go further in the study of bone context impact, we modified MM to create a “bone-like environment” under hypoxic condition (Reffuveille et al., 2018). Bone site and, more particularly, the low bone vascularization after surgery lead to nutrient starvation. We found that the lack of amino acid increased biofilm proportion (biomass and adherent cells) for all strains, even without oxygen. This nutritional deficiency is known to be linked with pp(G)pp-mediated stress responses leading to increased biofilm formation (Nguyen et al., 2011; de la Fuente-Núñez et al., 2014). Then, bone tissue is the main reservoir of magnesium of the human body, and the infection causes the bone matrix resorption, which releases a local amount of this cation (Kratz et al., 2004; González et al., 2009; Wright and Nair, 2010). Combined with amino acids depletion, a high concentration of magnesium increased the biofilm biomass proportion, which suggested that bacteria adapted their matrix production. Nevertheless, without glucose, biofilm biomass revealed by crystal violet assay were reduced, with increased or similar proportion of adherent bacteria. This difference between biofilm biomass and adherent bacteria proportion could suggested that matrix formation was impacted. However, SEM acquisitions highlighted that CAA, not only glucose, was important for matrix production of CIP 53.154 strain under hypoxia. Indeed, both MSSA strains under CAA starvation with or without magnesium excess showed a similar aspect of a fibrous matrix but different from control. However, the bone-like environment condition enhanced a similar matrix production for all the studied strains. This particular condition was the only one to enhance matrix production for USA300 MRSA strain. MRSA strains are known to adopt biofilm lifestyle in response to stress such as antibiotic pressure (Mirani et al., 2015). We suggested that USA300 strain only produce matrix under stressful condition such as BLE.

Biofilm matrix in MM without oxygen was mainly composed of PIA and eDNA for CIP 53.154 strain, PIA for SH1000 strain, and eDNA for USA300. These results were sustained by another study comparing MSSA and MRSA biofilm matrix (McCarthy et al., 2015). Grown in a bone-like environment, the three-studied strains harbored the same phenotype regarding the matrix component proportions: less bacteria were quantified, and a drastic decrease in each component was noticed. MSSA strains produce equal amount of matrix components, but SH1000 strain showed a higher proportion of bacteria. USA300 strain built a matrix, which seemed to be mainly composed of eDNA and only few proteins. However, these results are contrasting with observation of biofilms through SEM. Indeed, SH1000 and USA300 strains did not seem to produce matrix under MM and hypoxic condition using SEM, whereas matrix components were detected by CLSM. We suggested that such differences could be explain by the fact that CLSM revealed matrix over and under bacteria, whereas SEM only revealed biofilm surface. Furthermore, preparations steps were not the same and could alter biofilm matrix. Thus, it is important to analyze the experiments altogether to better decipher our results, as crystal violet staining, counting method, and MEB acquisitions tend to lead to similar conclusions. Moreover, MEB images showed structures, which could be dying or dead bacteria providing matrix structures non-labeled by fluorochromes.

To investigate the impact of a bone-like environment on metabolic pathways and stress responses involved in biofilm formed by each strain, several gene expressions were monitored through RT-qPCR, comparing BLE to MM conditions. The matrix produced by biofilm-embedded bacteria in MM without oxygen was different for the three strains, and we expected to get different gene deregulations. Focusing on biofilm initiation by studying global stress response genes, the expression of *recA* and *lexA* was significantly increased for all strains, and the *rsh* gene expression was upregulated. Both enhancement of *recA*, inducer, and of l*exA*, repressor, was surprising regarding the fact that these proteins had an opposite regulator role on the SOS pathway. However, we speculated that the existence of a feedback loop could explain this phenomenon; stringent response encoded by *rsh* gene induced the expression of *recA*, and the overexpression of *recA* led to an overexpression of its regulator l*exA* (Podlesek and Žgur Bertok, 2020). In this same condition, *sarA* was overexpressed for all the three strains. *sarA* is required for several virulence factors transcription and is needed for biofilm formation (Yu et al., 2020). Taking together, these results suggested involvement of common stress responses for *S. aureus* biofilm establishment in a bone-like environment. This environmental stress was confirmed by increased proportion of damaged and dead bacteria for the three strains.

Adhesion is an essential step of biofilm formation for which FnbpB facilitates fibronectin binding (McCourt et al., 2014). Fibronectin rapidly recovers foreign material and lead to initial attachment of bacteria in prosthetic device infection (Buck et al., 2010). Only MSSA strains cultured in BLE displayed an enhanced *fnbpB* expression, which was probably linked to SOS induction (Bisognano et al., 2004). However, its expression was downregulated for MRSA, underlining another involved mechanism for this strain. These results are in accordance with the matrix protein proportion of MSSA strains, which is higher than for USA300 strain.

Then, maturation of biofilm through matrix production (among eDNA and exopolysaccharides components) is a key for biofilm formation. Contrary to CIP 53.154 biofilm under aerobic condition, *icaC* expression was not modified in bone-like environment for MSSA strains and even deregulated for MRSA. However, under hypoxic condition, a study has shown that *srrA* induces PIA production (Kinkel et al., 2013). Unfortunately, even if *srrA* was upregulated for the three strains, PIA quantification through confocal microscopy revealed that this polysaccharide level was reduced into a bone-like condition for MSSA, and MRSA did not seem to produce any PIA. This is consistent with the fact that glucose supply was limited by MM modification (no Gluc), limiting saccharide source to produce PIA. Further investigations are needed to explain these results.

The proportion of eDNA in the matrix could be linked to *cidA* and *nuc* expressions. Interestingly, the expression of these two genes was variable among the three strains. We suggested that 24-h-old biofilms grown in a static assay were composed of bacteria in different states, and dispersal mechanisms could be engaged. However, the downregulation of *cidA* in SH1000 and USA300 corresponded to a decrease in eDNA level under confocal microscopy. It is interesting to notice that CIP 53.154 matrix decrease in bone-like environment could be linked to an upregulation of *nuc*.

Altogether, our results showed that both MSSA and MRSA responded to bone-like environment, increasing their biofilm biomass and number of adherent cells proportion but with heterogeneous matrix components and production. Moreover, we speculated about a universal response to bone-like environment through stringent and SOS response but also a preferential expression of *sarA* and *srrA* biofilm inducers. Moreover, all strains presented high dead or damaged bacteria level, with eDNA as the main matrix component. The description of this matrix and embedded bacteria will allow the selection of antibiofilm molecules against the biofilm tolerance and persistence in the specific bone context.

To develop future strategies for the prevention of biofilm formation in the bone environment, matrix eDNA seems to be a valid target to weaken the biofilm structure and disturb its formation and maturation. Furthermore, targeting stress response such as SOS-mediated response or global regulatory protein such as SarA could be an interesting approach, but further investigations are needed, by using knockout or suppression mutants for example. Finally, the model developed here will allow the study of clinical strains. In addition, it is important to remember that in this study, we used extreme conditions to be closer to the bone environment but which remained far from the reality of what is occurring *in vivo*. However, our results underlined the need to change the classical laboratory media used (like nutritive medium or brain heart infusion) for more realistic conditions to understand the “real-life” phenomena.

## Conclusion

In conclusion, we have studied three strains whose *in vitro* biofilms were very different from each other, involving several molecular mechanisms. However, factors mimicking bone microenvironment triggered common responses in these bacteria and lead them to develop more similar biofilms. The identification of host factors that induce biofilm formation will be essential. Targeting these mechanisms is the key in the battle against infection-related biofilms. This study underlined the strain-specific response to the same conditions and the urgent need to develop adapted *in vitro* models to properly screen antibiofilm molecules.

## Data Availability Statement

The raw data supporting the conclusions of this article will be made available by the authors, without undue reservation.

## Author Contributions

FL, FR, and SG designed the research. FL and JV-S performed the experiments. FV performed SEM images. CT provided methodologies to analyze CLSM acquisitions. FL, FR, FV, CM, and CT analyzed the data. FL, SG, FR, and MC wrote the manuscript. All authors reviewed the manuscript.

## Conflict of Interest

The authors declare that the research was conducted in the absence of any commercial or financial relationships that could be construed as a potential conflict of interest.

## Publisher’s Note

All claims expressed in this article are solely those of the authors and do not necessarily represent those of their affiliated organizations, or those of the publisher, the editors and the reviewers. Any product that may be evaluated in this article, or claim that may be made by its manufacturer, is not guaranteed or endorsed by the publisher.
