# Supplementary material for: Staphylococcus aureus Strain-Dependent Biofilm Formation in Bone-Like Environment
Source: Front Microbiol. 2021 Sep 7;12:714994. doi: 10.3389/fmicb.2021.714994 (PMC8453086; doi:10.3389/fmicb.2021.714994)
Supplement: Supplementary file 1 [file Data_Sheet_1.docx]

Supplementary Material

**Supplementary data S1.** Raw data of planktonic growth, biofilm biomass and number of live adherent bacteria. Biofilms were grow in minimal medium (MM) or modified minimal medium (no CAA, without casamino acids; 10x Mg, x10 minimal medium concentration; no Gluc, without glucose) with (white histograms) or without (black histograms) oxygen for 24 hours before absorbance measurement and live adherent bacteria numeration. (A) Results represent the absorbance at 600 nm (planktonic growth) before crystal violet staining. (B) Results represent the absorbance at 595 nm (biofilm biomass) after crystal violet coloration. Experiments were performed at least four independent times with three technical replicates. (C) Results represent the number of live bacteria adherent on Thermanox^TM^ coverslips. Experiments were performed at least four independent times with two technical replicates. Error bars represent standard errors for each average value. Statistical analyses were performed using the exact non-parametric Wilcoxon Mann Whitney test for independent samples: ¤, ¤¤, ¤¤¤, ¤¤¤¤ Statistically significantly different from aerobic control (*p<*0.05; *p<*0.01; *p<*0.001; *p<*0,0001); *, **, *** Statistically significantly different from anaerobic control (*p<*0.05; *p<*0.01; *p<*0.001).
